# Clinicopathological Characteristics and Influencing Factors of Renal Vascular Lesions in Anti-neutrophil Cytoplasmic Autoantibody-Related Renal Vasculitis

**DOI:** 10.3389/fmed.2021.710386

**Published:** 2021-09-28

**Authors:** Ruiqiang Wang, Yunqi Wu, Xiaofeng Zhang, Dongyue An, Ningning Guo, Yuanyuan Guo, Jin Wang, Lin Tang

**Affiliations:** Department of Nephrology, The First Affiliated Hospital of Zhengzhou University, Zhengzhou, China

**Keywords:** anti-neutrophil cytoplasmic autoantibody-related renal vasculitis, renal vascular lesions, clinicopathological characteristics, influencing factors, prognosis

## Abstract

The purpose of this study was to evaluate the clinicopathological features of different degrees of extraglomerular renal vascular lesions (RVLs) in patients with anti-neutrophil cytoplasmic antibody (ANCA)-associated renal vasculitis and explore their clinical determinants. This is a retrospective study of 186 patients with ANCA-associated renal vasculitis diagnosed at the First Affiliated Hospital of Zhengzhou University from January 2014 to April 2019. The patients who met the inclusion criteria were divided into non-renal RVLs, mild RVLs, moderate RVLs, and severe RVLs. It was found that there were significant differences in serum creatinine (SCR), estimated glomerular filtration rate (eGFR), erythrocyte sedimentation rate (ESR), high-density lipoprotein (HDL), systolic blood pressure (SBP), the prevalence rate of hypertension, the proportion of normal glomeruli, and the proportion of sclerotic glomeruli and interstitial fibrosis integral. SCR and ESR are independent risk factors for RVLs. The participants were followed up for 1 year, and the progression to end-stage renal disease (ESRD) and death was defined as endpoint events. We found that the survival rate of patients without RVLs was significantly higher than that of patients with RVLs and that the RVLs were an independent risk factor for ESRD or death. Early intervention in the progression of RVLs can improve the prognosis.

## Background

Anti-neutrophil cytoplasmic antibody (ANCA)-associated vasculitis (AAV) refers to a group of multisystem disorders characterized by necrotizing inflammation and destruction of small- and medium-sized blood vessels in conjunction with ANCA. The kidney is the most common organ involved in AAV, and more than 75% of patients with AAV have renal involvement and missed or delayed diagnosis may endanger the life of a patient (1–3). In 2010, a new histopathological classification was proposed, which included four major categories, foci, mixed, crescent colored, and sclerosis, and provided valuable information to clinicians (4). However, the classification is based only on glomerular lesions, and parameters of renal interstitial vascular lesions are excluded. As for most individuals with kidney diseases, the vascular component plays a second significant role in the disease process (5). However, AAV involves systemic small vessels, and it is also important to assess the damage to extraglomerular vascular lesions. In this retrospective observational study, we indicated and graded the severity of vascular lesions using a semi-quantitative scoring system (6) and further assessed their associations with clinical and pathological indexes and their influencing factors in patients with ANCA-associated renal vasculitis, providing a reference for delaying the progression and improving prognosis.

## Methods

### Study Participants and Data Collection

This study retrospectively enrolled 186 patients with ANCA-associated renal vasculitis admitted to the First Affiliated Hospital of Zhengzhou University between January 2014 and April 2019. The inclusion criteria were: (1) the presence of hematuria (>10/mm^3^) and/or proteinuria (>300 mg/day); (2) the detection of a positive ANCA by an antigen-specific immunoassay and/or indirect immunofluorescence; (3) the observation of more than 10 glomeruli in pathological sections, and (4) confirmation by renal biopsy of the presence of pauci-immune glomerulonephritis. The exclusion criteria were the following: (1) systemic diseases involving the kidneys, such as hepatitis B virus-associated glomerulonephritis, diabetic nephropathy, and systemic lupus erythematosus, and other primary glomerular diseases, such as membranous nephropathy and IgA nephropathy; (2) positive anti-glomerular basement membrane antibodies or renal pathological immunofluorescence showing a linear deposition of immunoglobulin or immunoglobulin deposition > 2+. The study was approved by the Committee of the First Affiliated Hospital of Zhengzhou University (Henan, China, No.2019-KY-015).

### Definition of Clinical and Laboratory Indicators

Clinical indicators included, age, gender, the prevalence of hypertension, diabetes mellitus (DM), cardiovascular diseases, systolic blood pressure (SBP), diastolic blood pressure (DBP), leukocyte count (WBC), hemoglobin (HB), platelet count (PLT), serum creatinine (SCR), serum uric acid (UA), estimated glomerular filtration rate (eGFR), serum albumin (ALB), 24 h-proteinuria (24 h-TP), urine RBC count, total cholesterol (T-CHO), triglycerides (TGs), high-density lipoprotein (HDL), low-density lipoprotein (LDL), C-reactive protein (CRP), erythrocyte sedimentation rate (ESR), complement C3, complement C4, immunoglobulin A (IgA), immunoglobulin G (IgG), immunoglobulin M (IgM), myeloperoxidase antibody (anti-MPO), protease 3 antibody (anti-PR3), and the Birmingham Vasculitis Activity Score (BVAS). eGFR was calculated with the Chronic Kidney Disease Epidemiology Collaboration (CKD-EPI) equation. Hypertension refers to a blood pressure ≥ 140/90 mmHg (7). DM is defined by the American Diabetes Association (ADA) as “Standards of Medical Care in Diabetes-2019.” Participants were identified as having DM if they had FPG ≥ 126 mg/dl (7 mmol/L) or 2 h PG ≥ 200 mg/dl (11.1 mmol/L) during a 75-g oral glucose tolerance test (OGTT), or glycosylated HB (HbA1c) ≥ 6.5%, random plasma glucose ≥ 200 mg/dl (11.1 mmol/L) in patients with classic symptoms of hyperglycemia or hyperglycemic crisis (8). Coronary artery disease refers to typical angina symptoms, ischemic ST-T changes in electrocardiograms, or a history of myocardial infarction (9).

### Histological Examination of Renal Biopsy Specimens

The presence of fibrous, cellular, and cellular fibrous crescents, fibrous necrosis, destruction of Bowman's capsule, and global glomerulosclerosis of each glomerulus was recorded and calculated as the percentage of the total number of glomeruli. The presence of microangiopathic lesions was calculated as the percentage of the number of renal specimens in the four groups. Interstitial fibrosis and tubular atrophy were evaluated according to the extent of interstitial fibrosis with tubular atrophy in the cortex: 0 = no or trivial interstitial fibrosis (<5% of unscarred parenchyma), 1 = 6–25% of interstitial fibrosis, 2 = 26–50% of interstitial fibrosis, and 3 = more than 50% of interstitial fibrosis. Interstitial inflammation was evaluated according to the extent of inflammatory cells in the cortex: 0 = no or trivial interstitial inflammation (<10% of unscarred parenchyma), 1 = 10–25% of parenchyma was inflamed, 2 = 26–50% of parenchyma was inflamed, 3 = more than 50% of parenchyma was inflamed (10). (1) Normal glomeruli are defined as slight changes caused by ischemia or a small amount of inflammatory cell infiltration (less than four monocytes, lymphocytes, or neutrophils) without vasculitis and glomerulosclerosis. (2) Global glomerulosclerosis means that more than 50% of glomerular clusters form scars in the sclerotic area. (3) Cellular crescent means that more than 50% of the crescent is occupied by cells. (4) Fibrous crescent means that more than 90% of the crescent is occupied by the extracellular matrix. (5) Cellular fibrous crescent means that <90% of the crescent is occupied by extracellular matrix and that <50% of the crescent is occupied by cells. (6) Interstitial inflammatory cell infiltration means excessive inflammatory cells in the interstitium of the renal cortex, excluding the subcapsular area and the area surrounding global glomerulosclerosis. (7) Interstitial fibrosis is defined as increased extracellular matrix separating tubules in the cortical area, excluding the subcapsular area (10). (8) Tubular atrophy is defined by a thick irregular tubule basement membrane with a decrease in the diameter of the tubules. It is scored according to the percent of the cortical area involved, excluding the subcapsular area (10). (9) Microangiopathic lesions include subintimal edema, endothelial cell swelling, small artery thrombosis, and/or fibrinoid necrosis that are considered acute. Arterial onion dermal lesions refer to the thickening of the fibrous intima, which appears as concentric circles and are considered chronic (11).

### Scores of Vascular Lesions

Kidney biopsy specimens were examined by light microscopy, electron microscopy, and immunofluorescence. These specimens were then reviewed and scored by two independent and experienced pathologists who did not know the clinical indicators of the patients. If any disagreements arose during the evaluation, a third pathologist was consulted and reached a final consensus. RVLs in this study referred to extraglomerular vascular lesions, including arterial fibrotic intimal thickening and arteriolar hyaline, whose scores were evaluated based on the definition described in the Oxford classification, with some modifications (12). The rules for scoring RVLs were as follows: (1) arterial vitreous lesions: the presence of vitreous lesions on the wall of an artery or small artery was scored as 1, while their absence was scored as 0; (2) arterial fibrotic intimal thickening: fibrotic intimal thickening was scored by comparing the thickness of the intima with that of the media in the same segment of the vessel: 0 = normal; 1 = less than the thickness of the media; 2 = exceeding the thickness of the media. The arterial fibrotic intimal thickening score for this specimen was based on the highest arterial score. Adding them together, the patients were divided into four groups: 0 = without RVLs; 1 = with mild RVLs; 2 = with moderate RVLs; and 3 = with severe RVLs. The score only quantitatively evaluates extraglomerular vascular lesions, even though the two lesions have different pathogenetic mechanisms.

### Statistical Analyses

Continuous variables that conformed to a normal distribution were expressed as mean ± SD, and those that were not normally distributed were expressed as median with interquartile range (IQR). Categorical variables were expressed as frequency and percentage. Comparisons of continuous variables among the four groups were determined by one-way ANOVA or Kruskal-Wallis H test. An χ^2^ test was performed to compare categorical variables between the groups. A binary logistic regression analysis model was used to analyze the factors influencing RVLs in patients with ANCA-associated renal vasculitis. The results were expressed as the ratio (OR) and 95% confidence interval (CI). Survival curves were plotted using the Kaplan-Meier (K-M) method, and differences in survival curves were compared by a Log-rank test. Univariate and multivariate COX regression models were used to identify the predictors of end-stage renal disease (ESRD) or death. The results were expressed as hazard ratio (HR) (with 95% CI). *p* < 0.05 was considered statistically significant. SPSS statistical software version 26.0 (SPSS 26.0) was used for statistical analysis.

## Results

### Correlations Between Vascular Lesions and Clinical Data

**Figure 1:**
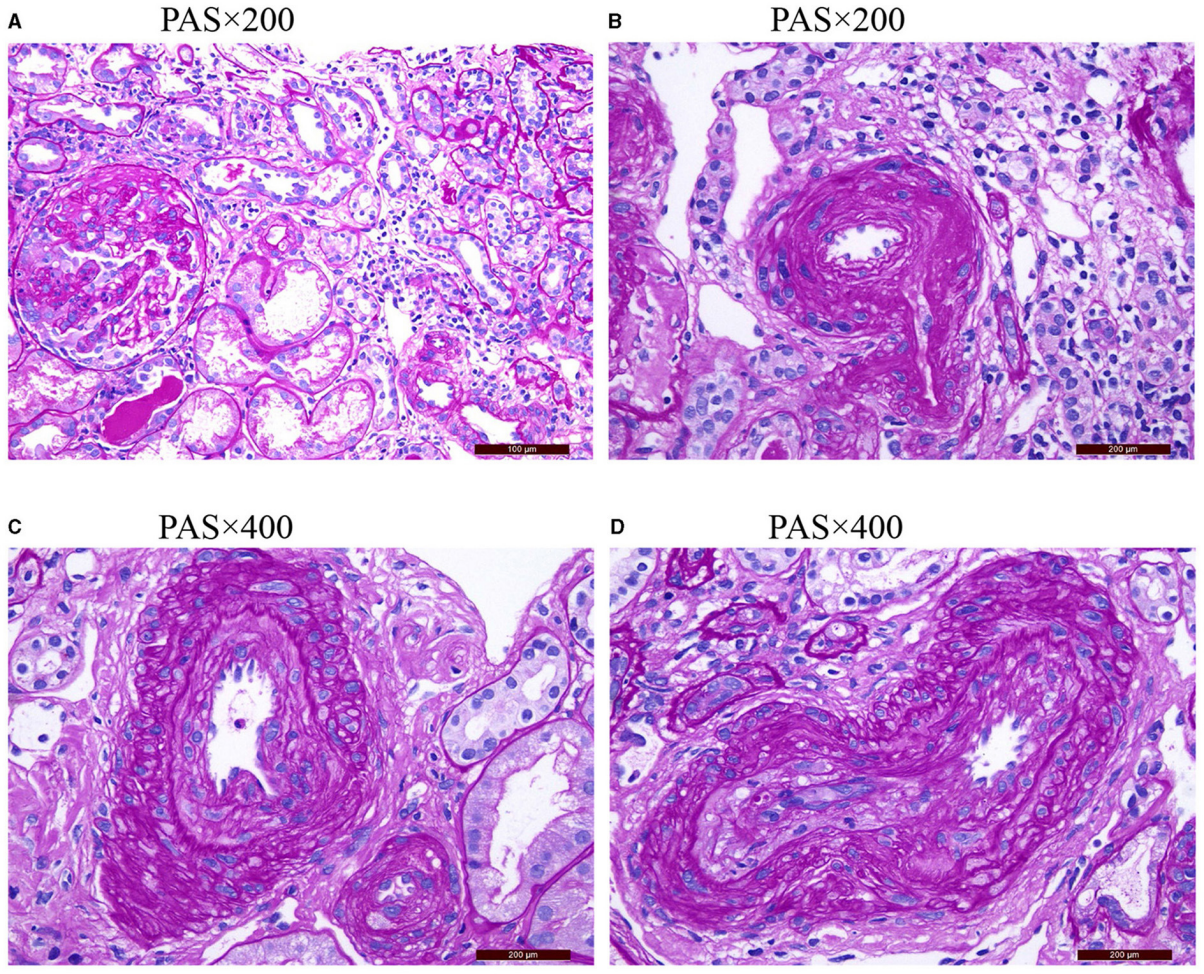
**(A)** Shows the normal appearance of extraglomerular vessels under a light microscope. **(B)** Shows the arteriolar hyaline. **(C)** Shows the intimal thickening of arterial fibrosis that does not exceed the media thickening of the same segment. **(D)** Shows that the intimal thickening of arterial fibrosis is greater than the media thickening of the same segment PAS, periodic acid-schiff stain.

One hundred eighty-six patients with ANCA-associated renal vasculitis were enrolled in this study. According to different degrees of vascular disease, they were divided into four groups: 23 cases (12.36%) in the non-RVL group, 72 cases (38.71%) in the mild RVL group, 65 cases (34.95%) in the moderate RVLs group, and 26 cases (13.98%) in the severe RVL group. Pathological manifestations of the RVLs are shown in Figure 1.

Of the 186 patients, 95 were men and 91 were women (16–82 years old). There were 84 (45.16%) with hypertension, 16 (8.6%) with DM, and 23 (12.37%) with cardiovascular diseases. The prevalence of hypertension was significantly higher in patients with severe vascular lesions than in patients without RVLs and with mild RVLs (*P* < 0.05). The SBP of patients with severe lesions was significantly higher than that of patients with mild and moderate RVLs and without RVLs (*P* < 0.05). The SCR and ESR of the patients with severe, moderate, and mild lesions were significantly higher than those of the patients without RVLs (*P* < 0.05), and eGFR was lower (*P* < 0.05).

There were no significant differences in age, gender, DBP, WBC, HB, PLT, UA, ALB, T-CHO, TG, LDL, 24 h-TP, urine RBC counts, CRP, complement C3, complement C4, IgA, IgG, IgM, anti-MPO, anti-PR3, BVAS, and the prevalence of DM and cardiovascular diseases among the patients in the four groups (Tables 1, 2).

**Table 1:** The general condition of the non-renal vascular lesion (RVL), mild RVL, moderate RVL, and severe RVL groups.

**Parameter**	**Non-RVLs**	**Mild RVLs**	**Moderate RVLs**	**Severe RVLs**	***P***
Age (years)	61 (47, 67)	62 (53, 67)	63 (53, 69)	61 (55, 70)	0.482
Duration of disease at diagnosis (month)	2 (0.5, 5.0)	1.5 (1, 4)	2 (1, 5.75)	2.5 (1, 7.5)	0.646
Gender (male/female, *n*)	12/11	37/35	33/32	13/13	0.999
Hypertension (*n*, %)	6 (26.09)^#^	27 (37.50)^#^	33 (50.77)	18 (69.23)	0.007
DM (*n*, %)	2 (8.70)	6 (8.33)	6 (9.23)	2 (7.69)	0.995
Cardiovascular diseases (*n*, %)	3 (13.04)	8 (11.11)	9 (13.85)	3 (11.54)	0.967

*P < 0.05 was considered significant. Compared with the severe RVL group:*

# *P < 0.05*.

**Table 2:** Clinical indexes of the non-RVL, mild RVL, moderate RVL, and severe RVL groups.

**Parameter**	**Non-RVLs**	**Mild RVLs**	**Moderate RVLs**	**Severe RVLs**	***P***
WBC (×10^9^/L)	7.73 (6.70, 10.4)	7.55 (6.23, 10.03)	8.20 (6.40, 11.05)	7.13 (6.02, 11.32)	0.673
HB (g/l)	94 (80, 110)	90 (78, 99)	85 (73.5, 102.0)	88.05 (75.50, 95.48)	0.289
PLT (×10^9^/L)	227 (193, 291)	252.5 (186.00, 356.75)	244 (189.5, 327.5)	253.5 (191.75, 308.75)	0.96
ALB (g/L)	32.6 (28.5, 39.1)	16 (12.0, 22.5)	17 (13, 21)	15 (11.75, 21.25)	0.356
ALT (U/L)	13 (9, 18)	12.5 (8, 23)	12 (8.0, 21.5)	9.5 (7.00, 18.25)	0.655
AST (U/L)	15 (12, 21)	31.6 (28.4, 35.8)	31.4 (28.1, 34.0)	30.5 (26.8, 36.6)	0.798
UA (μ mol/L)	302 (261, 438)	337.5 (254.25, 429.5)	354 (290, 443)	414.5 (294.25, 501.25)	0.294
SCR (μ mol/L)	144 (94, 200)	221.5 (152.25, 450.25)^*^	339 (171.5, 503.0)^*^	433 (237.15, 653.25)^*^	0.000
eGFR [ml/ (min·1.73m^2^)]	40 (25.16, 68.17)	22.98 (10.30, 41.73)^*^	15 (8.129, 33.325)^*^	12.185 (7.758, 23.038)^*^	0.000
T-CHO (mmol/L)	4.93 (3.82, 5.68)	4.24 (3.53, 5.09)	4.06 (3.27, 4.93)	4.15 (3.61, 5.16)	0.295
TG (mmol/L)	1.11 (0.94, 1.34)	1.09 (0.88, 1.56)	1.18 (0.82, 1.5)	1.31 (1.08, 1.83)	0.294
HDL (mmol/L)	1.26 (0.98, 1.48)	1.08 (0.83, 1.37)	0.98 (0.82, 1.18)	0.97 (0.80, 1.19)	0.04
LDL-C (mmol/L)	3.21 (2.06, 3.66)	2.69 (2.06, 3.31)	2.34 (1.79, 3.29)	2.48 (1.95, 3.02)	0.23
SBP (mmHg)	130 (120, 160)	135 (120, 169)^#^	156 (125, 180)^#^	180 (147, 190)^*^	0.000
DBP (mmHg)	90 (81, 100)	90 (84, 98)	90 (85, 100)	94.5 (89, 100)	0.115
Urine RBC counts (RBCs/HPF)	132 (33, 395)	160 (22, 313)	110 (24, 359)	182 (100, 443)	0.425
24 h-TP (g)	1.7 (1.19, 3.14)	1.41 (0.93, 2.30)	2.03 (0.88, 2.47)	2.08 (1.15, 2.54)	0.475
CRP (mg/L)	11.3 (1.47, 50.6)	32.02 (4.37, 90.61)	30.41 (6.05, 80.25)	36.40 (5.95, 70.98)	0.266
ESR (mm/h)	39 (25, 85)	87 (49, 111)^*^	80 (37, 117)^*^	99 (50, 121)^*^	0.006
Complement C3 (g/L)	0.97 (0.91, 1.13)	1.07 (0.91, 1.28)	1.06 (0.83, 1.17)	1.08 (0.78, 1.28)	0.499
Complement C4 (g/L)	0.28 (0.22, 0.29)	0.28 (0.24, 0.35)	0.26 (0.19, 0.30)	0.29 (0.19, 0.32)	0.287
IgA (g/L)	2.58 (1.62, 3.03)	2.47 (1.90, 3.03)	2.76 (1.96, 3.08)	2.95 (1.85, 3.16)	0.813
IgG (g/L)	13.4 (9.26, 15.29)	13.78 (10.23, 15.44)	13.78 (11.63, 16.35)	13.42 (11.38, 16.30)	0.529
IgM (g/L)	1.19 (0.78, 1.66)	1.19 (0.82, 1.39)	1.13 (0.9, 1.3)	1.19 (0.76, 1.56)	0.931
BVAS	15 (14, 21)	18 (16, 21)	18 (16, 21)	18.5 (16.5, 21)	0.35
Anti-MPO (U/mL)	583 (209, 900)	615 (225, 882)	512 (215.5, 863.5)	664.5 (169, 1136.75)	0.651
Anti-PR3 (U/mL)	8 (5, 15)	6.5 (4, 11)	7 (5, 11)	7 (3, 14)	0.861

*P < 0.05 was considered significant. Compared with the non-RVL group:*

* *P < 0.05; compared with the severe RVL group:*

# *P < 0.05*.

### Correlations Between Vascular Lesions and Pathological Data

The proportion of normal glomeruli of the patients with severe RVLs was significantly lower than that of the patients with mild or moderate and without RVLs (*P* < 0.05). The proportion of global glomerulosclerosis of the patients with severe RVLs was significantly higher than that of the patients with mild RVLs and without RVLs (*P* < 0.05) (Table 3). The renal interstitial fibrosis integral of the patients with severe RVLs was significantly higher than that of the patients with mild RVLs and without RVLs (*P* < 0.05) (Table 4). In addition, there were two cases (8.7%) with microangiopathic lesions in the without RVL group, which presented with fibrinoid necrosis and arteriolar thrombosis, respectively. The four cases (5.56%) in the mild RVL group were presented with fibrinoid necrosis. There were 10 cases (15.38%) in the moderate RVL group, namely, eight cases of fibrinoid necrosis and two cases of arteriolar thrombosis. There were five cases (19.23%) in the severe RVL group that included four cases of fibrinoid necrosis and a case of arteriolar thrombosis. No significant difference was found among the four groups.

**Table 3:** Pathologic characteristics of glomeruli of the non-RVL, mild RVL, moderate RVL, and severe RVL groups.

**Parameter**	**Non-RVLs**	**Mild RVLs**	**Moderate RVLs**	**Severe RVLs**	***P***
Normal glomeruli (%)	47.06 (16.67, 64.71)^#^	32.46 (15.38, 55.12)^#^	32 (14.16, 56.73)^#^	14.58 (9.38, 32.44)	0.008
Global glomerulosclerosis (%)	0 (0, 9.09)^#^	3.77 (0, 12.32)^#^	5.26 (0, 15.67)	8.58 (5.48, 32.86)	0.022
Rupture of Bowman's capsule (%)	20 (0, 35.71)	28.57 (12.5, 39.62)	18.75 (9.29, 28.34)	21.13 (11.94, 32.32)	0.146
Cellular crescent (%)	11.76 (0, 23.53)	7.14 (0, 24.87)	7.14 (0.85, 25.32)	9.41 (5.03, 26.85)	0.511
Cellular fibrous crescent (%)	3.03 (0, 25)	5.89 (0, 17.18)	2.38 (0, 12.25)	8.12 (0, 20.21)	0.332
Fibrous crescent (%)	11.76 (0, 21.95)	26.79 (4.16, 49.11)	18.60 (0, 50.71)	23.39 (3.95, 54.51)	0.395

*P < 0.05 was considered significant. Compared with the severe RVL group:*

# *P < 0.05*.

**Table 4:** Pathologic variables of renal tubulointerstitial tissue of the non-RVL, mild RVL, moderate RVL, and severe RVL groups.

**Parameter**	**Non-RVLs**	**Mild RVLs**	**Moderate RVLs**	**Severe RVLs**	***P***
Tubular atrophy integral	0 (0, 1)	0 (0, 1)	0 (0, 1)	1 (0, 1.25)	0.05
Interstitial inflammatory cell infiltration integral	2 (1, 3)	3 (2, 3)	3 (2, 3)	3 (2, 3)	0.062
Interstitial fibrosis integral	0 (0, 2)^#^	1 (0, 2)^#^	1 (0, 3)	2 (1, 3)	0.002
Microangiopathic lesions (%)	2 (8.70%)	4 (5.56%)	10 (15.38%)	5 (19.23%)	0.155

*P < 0.05 was considered significant. Compared with the severe RVLs group:*

# *P < 0.05*.

### Influencing Factors of RVLs in Patients With AAV-Related Renal Vasculitis

The patients were divided into two groups according to the presence or absence of RVLs. Univariate analysis showed that SCR, eGFR, SBP, ESR, tubular atrophy integral, interstitial inflammatory cell infiltration integral, and interstitial fibrosis integral were all influencing factors of RVLs (Table 5). Combined with the results of the univariate analysis and clinic practice, age, HB, SCR, HDL, 24 h-TP, CRP, ESR, BVAS score, normal glomeruli proportions, tubular atrophy integral, interstitial inflammatory cell infiltration integral, and interstitial fibrosis integral were included in multivariate binary logistic regression. Among the 186 patients with AAV, 23 (12.37%) had no RVLs, and 163 (87.63%) had RVLs. Likelihood ratio test (Wald χ^2^ = 33.307, *P* = 0.001) and goodness of fit tests (Pearson χ^2^ =3.644, *P* = 0.888), suggested that the binary logistic regression model was suitable for this study. The results showed that SCR and ESR had a significant positive correlation on RVLs in patients with AAV-related renal vasculitis (OR = 1.006, 95% CI: 1.001–1.011, *P* = 0.028; OR = 1.021, 95% CI: 1.005–1.038, *P* = 0.012) (Table 6).

**Table 5:** Results of the univariate analysis of RVLs.

**Parameter**	**OR**	**95% CI**	***P***
Gender (female)	1.051	0.439–2.52	0.91
Age	1.028	0.996–1.061	0.096
WBC	1.005	0.938–1.076	0.892
HB	0.98	0.958–1.002	0.081
PLT	1	0.996–1.004	0.99
ALT	1.006	0.968–1.047	0.746
AST	1.015	0.97–1.061	0.489
ALB	1.002	0.985–1.02	0.791
UA	1.001	0.997–1.005	0.57
SCR	1.006	1.002–1.01	0.000
eGFR	0.971	0.956–0.987	0.000
T-CHO	0.78	0.563–1.08	0.141
TG	1.449	0.668–3.143	0.288
HDL	0.332	0.147–0.747	0.008
LDL	0.788	0.543–1.145	0.22
SBP	1.017	0.999–1.034	0.047
DBP	1.015	0.973–1.059	0.488
Urine RBC counts	1	0.999–1.001	0.935
24 h-TP	0.953	1.007–0.794	1.278
CRP	1.01	0.998–1.021	0.068
ESR	1.02	1.008–1.033	0.001
Complement C3	1.186	0.23–6.117	0.838
Complement C4	22.822	0.124–4197.222	0.221
IgA	1.204	0.752–1.926	0.874
IgG	1.074	0.965–1.194	0.173
IgM	0.956	0.47–1.945	0.902
BVAS	1.079	0.972–1.197	0.151
Anti-MPO (+)	1.378	0.369–5.152	0.643
Anti-PR3 (+)	1.303	0.282–6.025	0.727
Hypertension (yes)	2.047	0.799–5.239	0.124
Diabetes mellitus (yes)	0.987	0.209–4.65	0.986
Cardiovascular diseases (yes)	1.04	0.285–3.794	0.952
Duration of disease at diagnosis	1.032	0.951–1.121	0.4
Normal glomeruli proportions	0.986	0.971–1.002	0.083
Global glomerulosclerosis proportions	1.03	0.989–1.072	0.104
Destruction of Bowman's capsule proportions	1.015	0.988–1.042	0.259
Cellular crescent proportions	0.998	0.977–1.02	0.889
Cellular fibrous crescent proportions	0.985	0.954–1.016	0.34
Fibrous crescent proportions	1.015	0.996–1.034	0.11
Tubular atrophy integral	2.005	0.965–4.163	0.037
Interstitial inflammatory cell infiltration integral	1.739	1.162–2.603	0.008
Interstitial fibrosis integral	1.692	1.082–2.647	0.014

**Table 6:** Logistic regression analysis of multiple factors in the occurrence of RVLs.

**Parameter**	**OR**	**95% CI**	***P***
Age	1.014	0.975–1.055	0.48
HB	1.013	0.982–1.045	0.406
SCR	1.006	1.001–1.011	0.028
HDL	0.512	0.185–1.417	0.198
24 h-TP	1.057	0.777–1.439	0.723
CRP	1.003	0.989–1.016	0.678
ESR	1.021	1.005–1.038	0.012
BVAS	1.02	0.902–1.152	0.753
Normal glomeruli proportions	1.004	0.983–1.025	0.715
Tubular atrophy integral	0.938	0.388–2.272	0.888
Interstitial inflammatory cell infiltration integral	0.844	0.471–1.515	0.571
Interstitial fibrosis integral	1.699	0.906–3.187	0.098

**Figure 2:**
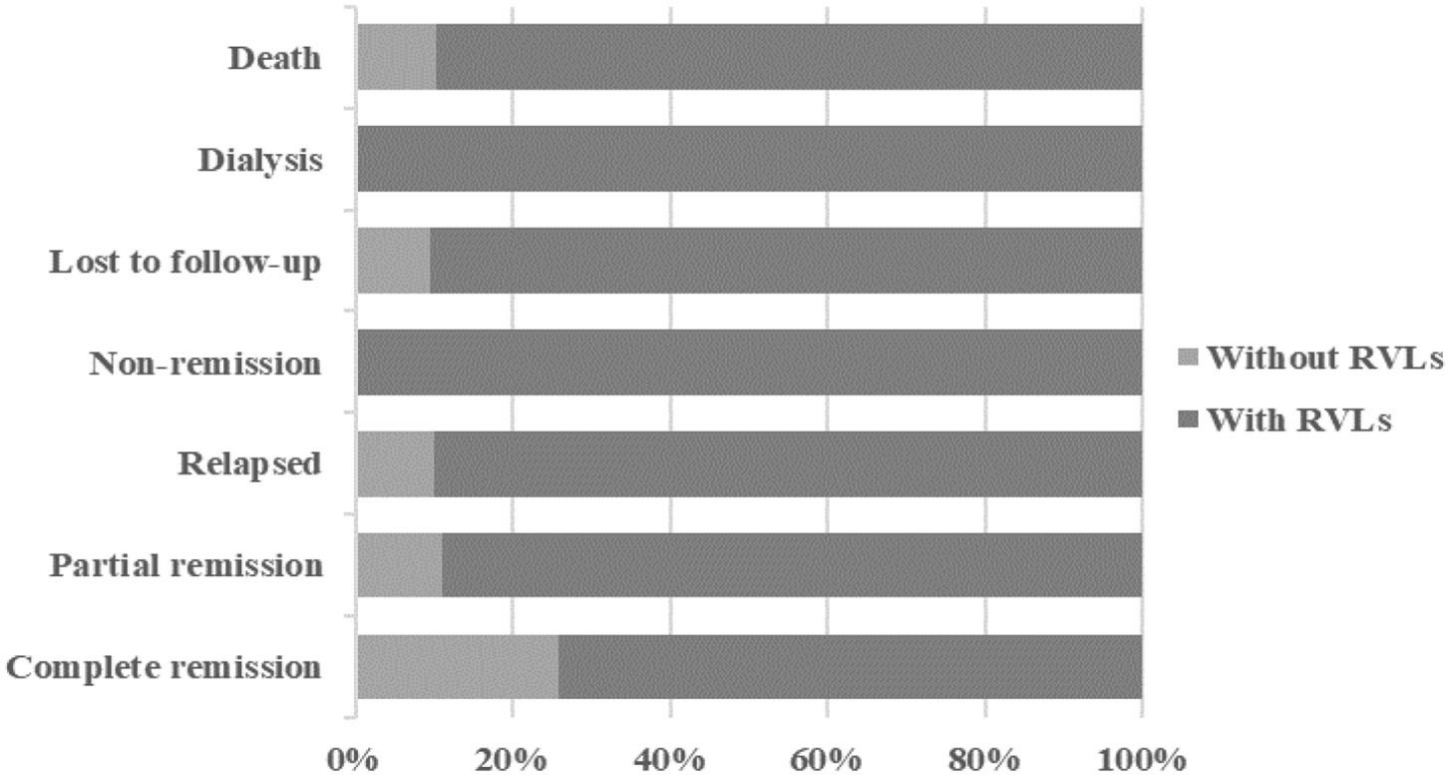
The outcome of the patients in two groups after a 1-year follow-up.

**Figure 3:**
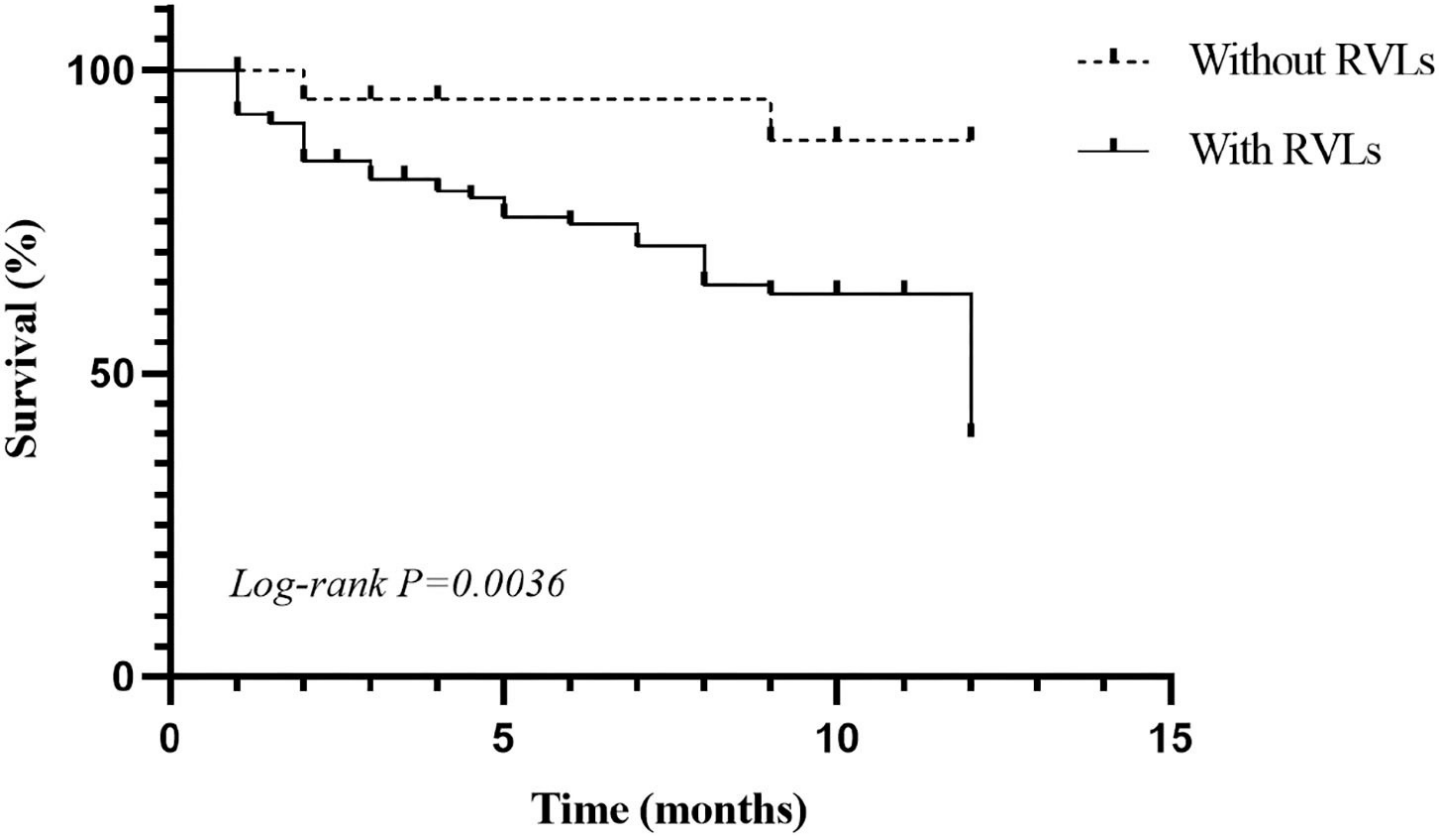
Kaplan–Meier (K-M) survival curve for anti-neutrophil cytoplasmic antibody (ANCA)-associated renal vasculitis.

During the 1-year follow-up, 54 of the patients were in complete remission, 9 were in partial remission, 10 relapsed after remission, 3 were in no remission, 42 were lost to follow-up, 39 underwent dialysis, and 29 died (Figure 2). We defined progression to ESRD and death or death as the endpoint event, and the K-M survival curve showed that RVLs were a predictor of poor prognosis in patients with AAV-related renal vasculitis (Figure 3). A univariate Cox proportional hazard regression model analysis revealed that PLT, SCR, UA, eGFR, SBP, DBP, urine RBC counts, complement C3, BVAS, RVLs, normal glomeruli proportions, cellular fibrous crescent proportions, fibrous crescent proportions, tubular atrophy integral, interstitial inflammatory cell infiltration integral, and interstitial fibrosis integral were associated with ESRD or death. A multivariate Cox proportional hazard regression model analysis showed that UA (HR = 1.003, 95% CI: 1.001–1.004, *P* = 0.002), urine RBC counts (HR = 1.001, 95% CI: 1–1.002, *P* = 0.008), BVAS (HR = 1.152, 95% CI: 1.058–1.253, *P* = 0.001), and RVLs (HR = 6.6623, 95% CI: 1.068–41.573, *P* = 0.042) were independent risk factors for ESRD or death in patients with ANCA-related renal vasculitis (Table 7).

**Table 7:** Cox proportional hazard regression model describing the long-term risk of end-stage renal disease (ESRD) or death in patients with ANCA-related renal vasculitis.

**Parameter**	**Univariate analysis**		**Multivariate analysis**	
	**HR (95% CI)**	***p***	**HR (95% CI)**	***P***
HB	0.996 (0.982–1.009)	0.537	1.016 (0.996–1.035)	0.114
PLT	0.997 (0.994–0.999)	0.007	0.998 (0.995–1.001)	0.165
SCR	1.002 (1.002–1.003)	0.000	1.002 (0.999–1.004)	0.124
UA	1.004 (1.002–1.006)	0.000	1.003 (1.001–1.004)	0.002
eGFR	0.966 (0.948–0.983)	0.000	1.019 (0.992–1.046)	0.165
ALB	1.001 (0.995–1.007)	0.801	1.002 (0.994–1.009)	0.657
HDL	0.642 (0.338–1.218)	0.175	1.374 (0.621–3.041)	0.433
SBP	1.008 (1.002–1.045)	0.045	1.001 (0.986–1.015)	0.936
DBP	1.023 (1.002–1.045)	0.035	0.996 (0.962–1.032)	0.841
Urine RBC counts	1.001 (1.000–1.002)	0.008	1.001 (1.000–1.002)	0.008
24 h-TP	1.126 (1.026–1.237)	0.013	0.996 (0.881–1.125)	0.946
Complement C3	0.353 (0.130–0.962)	0.042	0.759 (0.21–2.738)	0.673
Complement C4	0.074 (0.003–1.872)	0.114	0.004 (0.000–0.473)	0.023
BVAS	1.121 (1.049–1.198)	0.001	1.152 (1.058–1.253)	0.001
RVLs (yse)	5.73 (1.397–23.505)	0.015	6.6623 (1.068–41.573)	0.042
Normal glomeruli proportions	0.971 (0.958–0.985)	0.000	0.983 (0.964–1.003)	0.088
Cellular fibrous crescent proportions	1.018 (1.000–1.037)	0.046	1.018 (0.988–1.049)	0.234
Fibrous crescent proportions	1.013 (1.004–1.022)	0.004	1.007 (0.993–1.022)	0.324
Tubular atrophy integral	1.477 (1.122–1.945)	0.005	1.253 (0.784–2.003)	0.346
Interstitial inflammatory cell infiltration integral	1.548 (1.111–2.156)	0.010	1.231 (0.782–1.938)	0.370
Interstitial fibrosis integral	1.31 (1.047–1.640)	0.018	0.742 (0.534–1.032)	0.076

## Discussion

This study investigated the clinicopathological features of RVLs and their influence factors in 186 patients with AAV with renal involvement. Among the 186 patients with AAV, 163 (87.63%) had RVLs. It suggested that RVLs were common pathological characteristics in the renal biopsies of patients with ANCA-associated renal vasculitis. Among the patients with RVLs in this study, 72 cases (38.71%) of mild RVLs, 65 cases (34.95%) of moderate RVLs, and 26 cases (13.98%) of severe RVLs were included.

Anti-neutrophil cytoplasmic antibody-associated vasculitis affects small vessels throughout the body and is associated with the presence of ANCA in serum. In addition to the traditional methods of phagocytosis, attacking and killing, neutrophil extracellular traps (NETs) are an important means of defense against pathogen invasion (13). Persistent and prolonged exposure of NETS to their contents disrupts tolerance to specific self-antigens, particularly myeloperoxidase and protease 3. These antigens are presented to CD4^+^T cells *via* dendritic cells, resulting in ANCA production. ANCA induces the over-activation of neutrophils, leading to the production of abnormal cytokines, accompanied by the release of reactive oxygen species and lytic enzymes and further formation of NET, which damages vascular endothelial cells. NETs are not only involved in ANCA-mediated vascular injury but also in the production of ANCA itself. Therefore, a vicious cycle of NET formation and ANCA production is thought to be involved in the pathogenesis of AAV (14). Moreover, in this study, the patients with RVLs were found to have a worse prognosis than those without RVLs after the 1-year follow-up. Therefore, the assessment of vascular lesions is an important factor for the assessment of AAV.

The SCR of patients with severe, moderate, and mild RVLs was significantly higher than that of patients without RVLs, and eGFR was lower. The results of the regression analysis showed that SCR was an independent risk factor for RVLs. It was probably that the AAV disease activity, rapid progression, and prolongation of the duration of AAV, autoimmune abnormality, inflammation, oxidative stress, hemodynamic changes, mechanical stretch, and other factors led to endothelial dysfunction, thus resulting in arterial intimal thickening, arteriolar hyaline, and thrombosis. The above processes interacted with each other, exacerbating the development of renal dysfunction, and renal function loss is accompanied by the intimal hyperplasia of renal arterioles (15).

This study found that the higher the score of RVLs, the faster the ESR, and that ESR was an independent risk factor for the occurrence of RVLs. It is well-known that ESR reflects disease activity in many autoimmune diseases. The possible mechanism was that AAV disease activity activated the complement system, led to increased inflammatory response, and simultaneously resulted in the increase in fibrinogens and other inflammatory biomarkers, all of which contributed to the development of AAV-related renal vasculitis. With the worsening of renal function, the metabolism and elimination of fibrinogen decreased gradually, leading to acceleration of ESR (16). On the other hand, renal dysfunction might directly cause an increase in inflammatory mediators *via* a mechanism of increasing oxidative stress that could lead to the accumulation of advanced glycation end products. Levels of these oxidation products increase as the glomerular filtration rate declines, which might lead to arterial intimal thickening and directly increased vascular permeability (17). In addition, inflammation also affects lipid levels, which has attracted more and more attention in atherosclerosis and other immune-mediated diseases (18, 19). Therefore, ESR can indirectly reflect the situation of RVLs to a certain extent, control inflammation in the early stage of the disease as far as possible, delay the progression of vascular disease, and reduce the probability of death or entering the dialysis stage.

As the main protective protein in serum, HDL is pivotal in the prevention of atherosclerosis and is also considered a good indicator of cardiovascular disease risk and AAV (18, 20). This study showed that the lower the HDL, the higher the occurrence and the greater the severity of RVLs. Meanwhile, HDL is a protective factor for RVLs. It has been proved that MPO selectively targets and oxidizes HDL-bound APO-A1, which makes HDL lose its ability to control cholesterol outflow, acyltransferase activation, anti-inflammation, and anti-apoptosis. The decrease of serum HDL level will lead to the decrease in the regulation of complement pathway activation, and at the same time, the oxidation of MPO causes the complete or partial loss of the anti-inflammatory ability of HDL, which may be the potential mechanism of the decrease of HDL level in the pathogenesis of AAV (18, 21). Recent findings indicated a harmful interaction between autoantibodies targeting HDL lipoproteins and their components and lipid profiles in rheumatoid arthritis and systemic lupus erythematosus, and it was closely related to disease activity, anti-HDL antibodies, lipoprotein functionality, lipid profiles, and antioxidant features (22–24). So, there might be a detrimental interaction between autoantibodies targeting HDL lipoproteins and their component sand lipid profiles in ANCA related renal vasculitis patients. The interaction is related to the HDL antioxidant dysfunction, lipid profiles, lipoprotein functional impairment, and ultimately vascular endothelial cells dysfunction. The HDL dysfunction associated with anti-HDL antibodies may not be a specific phenomenon of a single disease but rather a common feature of the immune-mediated disease (25).

Hypertension has long been known to play an important role in the development of kidney damage, and because of its prevalence in the general population, it remains the second leading cause of end-stage renal disease, after diabetes (26). The prevalence of hypertension was prominently higher in patients with severe RVLs than in patients without RVLs and with mild RVLs. The SBP of patients with severe RVLs was prominently higher than that of patients with mild and moderate RVLs, and that of patients without RVLs, and was a risk factor for RVLs. ANCA attacks the kidney, resulting in glomerular capillary loop necrosis and crescent formation, leaving the glomerulus in a state of high hemodynamics. Hypertension in the glomerular blood pressure leads to hypertension of the glomerular capillaries stretching, endothelial injury, and dysfunction (even endothelial disintegration), and increased glomerular filtration protein, leading to glomerular collapse, segmental necrosis, and glomerular sclerosis. Simultaneously, high hemodynamics can significantly dilate the glomeruli and stretch mesangial cells, increase the synthesis of collagen and fibronectin, buffer glomerular hypertrophy to some extent, reduce glomerular pressure, and increase glomerular compliance. The interaction of the above factors leads to renal arteriosclerosis and renal parenchyma ischemia, aggravates renal parenchyma pathological changes and renal function damage, and affects the interstitial part of the kidney further. Adaptive structural changes in response to hypertension, such as increased medial wall thickness and reduced lumen diameter, lead to increased vascular wall stress and, ultimately, hypoxic-ischemic injury to the glomerular and tubule-interstitial structures due to decreased tissue perfusion. Hypertension and kidney damage interact with each other, forming a vicious circle (27). Therefore, the control of blood pressure, especially SBP, can delay the progression of renal vascular disease to a certain extent, thus improving the prognosis of patients.

Kidney disease is common in AAV, and the typical renal presentation is rapidly progressive. Histologically, pauci-immune necrotizing crescentic glomerulonephritis is the most typical pathological characteristic, and histologic findings remain the gold standard for diagnosing patients within ANCA-associated renal vasculitis (4). Our results found that the more severe the RVLs, the lower the proportion of normal glomeruli, and that the higher the proportion of global glomerulosclerosis, the higher the scores of interstitial fibrosis. Meanwhile, tubular atrophy integral, interstitial inflammatory cell infiltration integral, and interstitial fibrosis integral were risk factors for RVLs. The possible reasons were as follows: in the progression of glomerular injury, inflammatory cells and mediators acted on glomeruli and tubulointerstitial tissue, leading to the infiltration of interstitial inflammatory cells and release of cytokines, thus causing renal tubular atrophy, interstitial fibrosis, and vascular disease in the end. The tubulointerstitial lesions led to ischemia and hypoxia in renal tissue and further aggravated the infiltration of interstitial inflammatory cells, released inflammatory cytokines and inflammatory mediators, and promoted fibroblast proliferation. All of these could cause the activation and apoptosis of renal tubular epithelial cells, tubular atrophy, and accelerate the progression of renal interstitial fibrosis. Therefore, the early control of vascular disease may delay the progression of ANCA-associated renal vasculitis to a certain degree.

To the best of our knowledge, there are only few investigations on RVLs in patients with AAV. However, as this is a retrospective study, some limitations should be addressed. On the one hand, this is a single-center study. Because of the low incidence of AAV, poor prognosis, and difficult clinical diagnosis, the sample size of this study is relatively small. On the other hand, we focused more attention on the clinicopathological features and influencing factors of RVLs in AAV, not taking into consideration the effect of drug therapy on the patients. Therefore, multi-center large sample studies are needed to reveal the correlation further, evaluate the prognosis, and carry out basic experiments to explore the specific mechanism of disease.

## Conclusion

In conclusion, the severity of RVLs could be reflected in clinical indexes such as SCR, eGFR, HDL, SBP, ESR, and the proportion of normal glomeruli, the proportion of global glomerulosclerosis, and the score of interstitial fibrosis in renal pathology. The probability of death or access to dialysis within 1 year in patients with RVLs was significantly higher than that in patients without RVLs, and the RVLs were an independent risk factor. Moreover, SCR, eGFR, SBP, ESR, tubular atrophy integral, interstitial inflammatory cell infiltration integral, and interstitial fibrosis integral were risk factors for the occurrence of RVLs. HDL was a protective factor, and SCR and ESR were independent risk factors for RVLs. Therefore, it is necessary to control blood pressure and inflammation, protect renal function, and regulate blood lipids, which could delay the progression of vascular disease to a certain extent and improve the prognosis of patients.

## Data Availability Statement

The original contributions presented in the study are included in the article/supplementary material, further inquiries can be directed to the corresponding author/s.

## Ethics Statement

The studies involving human participants were reviewed and approved by The Ethics Committee of the First Affiliated Hospital of Zhengzhou University. Written informed consent to participate in this study was provided by the participants' legal guardian/next of kin.

## Author Contributions

RW conceived the study. RW and YW initially drafted the manuscript, performed the statistical analyses, and gave critical inputs to the discussion and interpretation of the data. DA, NG, and YG collected and interpreted the data. XZ and JW were responsible for reviewing and scoring the specimens. RW and LT prepared, reviewed, and revised the manuscript. LT supervised the study further. All the authors contributed to and approved the final manuscript.

## Funding

Open access funding was provided by the 2016 Key Science and Technology Plan project of Henan province (162102310198).

## Conflict of Interest

The authors declare that the research was conducted in the absence of any commercial or financial relationships that could be construed as a potential conflict of interest.

## Publisher's Note

All claims expressed in this article are solely those of the authors and do not necessarily represent those of their affiliated organizations, or those of the publisher, the editors and the reviewers. Any product that may be evaluated in this article, or claim that may be made by its manufacturer, is not guaranteed or endorsed by the publisher.
